# New Concept of Colonoscopy Assisted by a Microwave-Based Accessory Device: First Clinical Experience †

**DOI:** 10.3390/cancers17071073

**Published:** 2025-03-22

**Authors:** Oswaldo Ortiz, Oriol Sendino, Silvia Rivadulla, Alejandra Garrido, Luz María Neira, Josep Sanahuja, Pilar Sesé, Marta Guardiola, Glòria Fernández-Esparrach

**Affiliations:** 1 Endoscopy Unit, Hospital Clínic, University of Barcelona (UB), 08036 Barcelona, Spain; oortiz@clinic.cat (O.O.); sendino@clinic.cat (O.S.); srivadul@clinic.cat (S.R.); sese@clinic.cat (P.S.); 2 Instituto de Investigaciones Biomédicas August Pi i Sunyer (IDIBAPS), 08036 Barcelona, Spain; 3 MiWEndo Solutions, 08021 Barcelona, Spain; agarrido@miwendo.com (A.G.); lmneira@clinic.cat (L.M.N.); marta@miwendo.com (M.G.); 4 Anesthesiology Department, Hospital Clínic, University of Barcelona (UB), 08036 Barcelona, Spain; sanahuja@clinic.cat; 5 Facultat de Medicina i Ciències de la Salut, University of Barcelona (UB), 08036 Barcelona, Spain; 6 Centro de Investigación Biomédica en Red de Enfermedades Hepáticas y Digestivas (CIBEREHD), 08036 Barcelona, Spain

**Keywords:** colonoscopy, microwaves, polyp miss-rate, colorectal cancer prevention

## Abstract

**Background/Objectives**: Colonoscopies have some limitations that result in a miss rate detection of polyps. Microwave imaging has been demonstrated to detect colorectal polyps based on their dielectric properties in synthetic phantoms, ex vivo tissues and in vivo animal models. This study aims to evaluate, for the first time, the feasibility, safety and performance of microwave-based colonoscopy for diagnosis of polyps in real-time explorations in humans. **Methods**: This was a single-center, prospective, observational study. Patients referred for diagnostic colonoscopy were explored with a device with microwave antennas which was attached to the tip of a standard colonoscope. The primary outcomes were rate of cecal intubation, adverse events, mural injuries and performance metrics for the detection of polyps. Secondary outcomes were the following: patients’ subjective feedback, procedural time and perception of difficulty according to the endoscopist. **Results**: Fifteen patients were enrolled. Cecal intubation rate was 100%, with a mean time of 12.7 ± 4.9 min (range 4–22). Use of the device did not affect the endoscopic image, and polypectomy was successfully performed in all cases. In on scale from zero (not difficult) to four (very difficult), the maneuverability during the insertion was considered ≤2 in the 86.7% (13/15) of colonoscopies. Only 16 incidents were reported in 14 patients: 11 (67%) superficial hematomas, 2 minor rectal bleedings, 1 anal fissure, 1 rhinorrhea and 1 headache. Most of the patients (94%) reported no discomfort or minimal discomfort before discharge (Gloucester score 1 and 2, respectively). In the six patients with 23 polyps used for the performance analysis, the sensitivity and specificity were 86.9% and 72.0%, respectively. **Conclusions**: microwave-based colonoscopy is safe and feasible and has the potential to detect polyps in real colonoscopies.

## Simple Summary

Colorectal cancer is one of the leading causes of cancer-related deaths worldwide, and its early detection is crucial for improving patient outcomes. Colonoscopy is the most effective screening tool, but still misses 22% of polyps or cancer precursors. This paper presents, for the first time, the safety and feasibility of a pioneering MiWEndo microwave endoscopy system. The MiWEndo System, a colonoscopy accessory, uses microwave signals to analyze tissue dielectric properties and identify polyps without modifying clinical practice. The sensitivity and specificity results (86.9% and 72%, respectively) position microwave endoscopy as a potential tool to complement and assist colonoscopy in detecting low-optical-contrast polyps, such as flat or more subtle lesions. Additionally, MiWEndo could provide valuable support in real-time clinical decision-making due to the negative predictive value of 97.3% for detecting adenomas.

## 1. Introduction

Colorectal cancer (CRC) is the third most common cancer worldwide and the second leading cause of death in men and women [1]. Colonoscopies have been demonstrated to prevent CRC by detection and by allowing for resections of precursor lesions [2]. Nevertheless, it is not a perfect technique, and post-colonoscopy CRC, defined as colorectal cancer diagnosed after a colonoscopy in which no cancer was detected and before the next recommended exam, remains a primary issue [3]. Post-colonoscopy CRC may arise from possible missed lesions on index procedures, accounting for 70–80% of post colonoscopy CRC [4,5]. This inefficiency of colonoscopies can be partially explained by human limitations such as distractions, fatigue, shorter withdrawal time, incomplete colonoscopies or inadequacies in the inspection technique, but also by visual limitations, since some lesions are beyond the endoscope field of vision (<180°), mainly due to angulations, folds, heterogeneous illumination and poor bowel cleansing.

To overcome these limitations, several devices have been developed to improve image definition, retrovision capability or mucosal flattening [6]. However, they do not allow total exposure of colonic mucosa. More recently, artificial intelligence (AI) has irrupted as an alternative to improve the outcomes of colonoscopy and has been studied in a range of clinical applications, showing improved overall polyp detection rates [7,8,9].

Microwave imaging (MWI) is a promising technology that allows for a 360° view of the mucosa, reducing these visual limitations. MWI is based on the detection of changes in the dielectric properties of biological tissues, properties that are determined by the water content of the tissues [10,11,12,13]. Since microwave imaging (MWI) uses low-power, non-ionizing radiation, it poses no risk of thermal injury or other biological effects on the patient. We have developed a microwave endoscopy system intended to be used for assistance in conventional colonoscopes for polyp detection, and we have successfully completed all electrical safety and electromagnetic compatibility tests in accordance with IEC 60601 and verified that the specific absorption rate (SAR) levels comply with the ICNIRP (International Commission on Non-Ionizing Radiation Protection) standards [14,15]. The principle of functionality of this microwave-based device relies on the fact that adenomatous polyps and cancer have increased vascularization due to neoangiogenesis and, therefore, greater water content that translates into higher dielectric properties in comparison with healthy colonic mucosa [16]. Therefore, and differently from other technologies, microwave-based colonoscopy does not depend on the optical endoscopic image. So far, this technology has demonstrated its diagnostic capability in previous preclinical studies with phantoms [17], ex vivo human colon samples [18]) and in vivo studies with porcine tissues [19].

In this single-center pilot study, we aimed to investigate, for the first time, the feasibility, safety and performance of colonoscopies assisted by a microwave-based accessory device. Secondary objectives were to assess the perception of difficulty based on the endoscopist and the patient’s comfort. Preliminary data were presented as a poster at DDW 2023 and ESGE Days 2023 [20,21].

## 2. Materials and Methods

### 2.1. Patient Selection and Study Design

This was a prospective, observational, single-center non comparative study performed at a tertiary center (Hospital Clinic Barcelona). The study protocol was approved by the local ethical committee (HCB/2022/0690) and Spanish competent authority (1023/22/EC-R) and registered in clinical trials (NCT05477836) before the initiation of inclusion. The consent form for participation was distributed to all participants and signed.

Eligible participants were patients who met the following inclusion criteria: (a) age ≥ 50 years referred for a diagnostic colonoscopy for symptoms (anemia, hematochezia/rectorrhagia, abdominal pain, diarrhea, constipation) and/or post-polypectomy surveillance and (b) written informed consent. Exclusion criteria were patients in whom the possibility of performing a complete colonoscopy was reduced due to known colonic strictures, recent acute diverticulitis episodes, inflammatory bowel disease or previous incomplete colonoscopy; suspected or proven lower gastrointestinal bleeding; non-correctable coagulopathy; ASA IV patients, urgent colonoscopy and inadequate bowel cleansing.

### 2.2. Microwave Imaging Description

**Figure 1:**
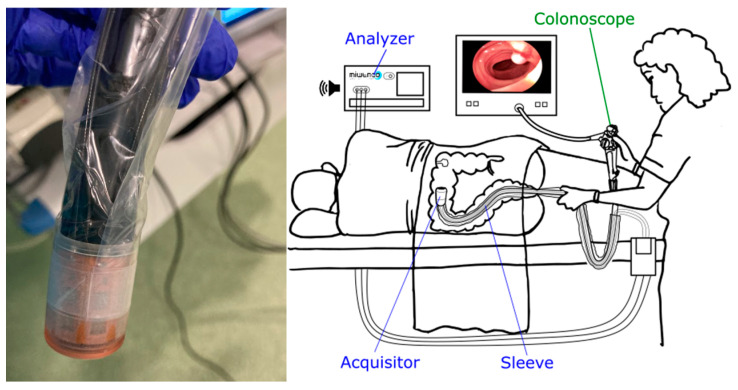
The MiWEndo system comprises two main components: a cylindrical ring-shaped acquisition device (Acquisitor) that is attached to the colonoscope’s tip, and an external unit (Analyzer) that is connected to the Acquisitor via cables. The Analyzer contains a microwave transceiver and a processing unit.

MiWEndo (MiWEndo Solutions SL, Barcelona, Spain) is a disposable device that consists of two parts: (a) a cylindrical ring-shaped disposable part (the Acquisitor v22.1) measuring 30 mm in length and 20 mm in diameter that is attached to the tip of the colonoscope and (b) an external unit with a microwave transceiver and a processing unit (the Analyzer v22.1) [19]. The Acquisitor contains two rings with 8 antennas each (1 ring contains the transmitters and the other the receivers), connected via cables protected with a plastic sleeve to the Analyzer (Figure 1).

The MiWEndo System is based on MWI, which illuminates the colon with microwaves emanating from 8 antennas operating at 7.5 GHz [18] that cover the full perimeter of the colon. The 8 adjacent antennas collect the waves generated by their interaction with the colon, as detailed in [18]. In particular, the received field is measured at the receiving antenna adjacent to the active transmitting antenna and with the two closest diagonal antennas. The received field contains information regarding the spatial changes in the dielectric properties of the tissues. By processing this field with an imaging algorithm, the dielectric contrast of the colorectal tissues can be reconstructed. The resulting data represents a cross-sectional slice of the colon or rectum, referred to as a frame. As the colonoscope advances, the acquisition device continuously scans and processes the information at a rate of 5 frames/s (200 milliseconds per frame), ensuring full coverage of the colorectal lumen. These frames are analyzed by summing the magnitude of all their pixels, generating an image that maps dielectric property changes over time. Regions with brighter pixels indicate areas with a higher likelihood of containing a polyp.

### 2.3. Endoscopic Procedures

Anterograde cleansing was performed according to our center’s protocol. All patients were encouraged to undertake a diet low in fiber and fat for the 3 days before the procedure.

The procedures were performed with patients under sedation with propofol and remifentanil in perfusion administered by anesthesiologists. Bowel cleansing was considered adequate if the Boston score was ≥6 points (≥2 by colonic segment).

High definition (HD) colonoscopes (Olympus CF-HQ190L) were used in the study, and colonoscopies were performed by two experienced endoscopists previously trained on the use of the device. The MiWEndo device was attached to the tip of the scope and inserted through the colon. Carbon dioxide insufflation was used in all colonoscopies and the resection of all detected lesions was performed during the withdrawal. Cecal intubation was recognized through the usual landmarks (triradiate cecal folds, appendix orifice and ileocecal valve). After reaching the cecum and before starting withdrawal, the MiWEndo system was turned on. Therefore, during the withdrawal, each colonic segment was inspected with HD white light and MWI. Insertion time, withdrawal time excluding procedures and total procedure time were recorded with a stopwatch.

When a polyp was detected, features such as size in millimeters, morphology according to Paris classification [22] and location based on colonic segments (cecal, ascendant colon, hepatic flexure, transverse, splenic flexure, descendent colon, sigmoid, rectum) were collected. Resection techniques were applied based on the endoscopist’s judgment. Resected lesions were retrieved in separated containers and evaluated by an expert pathologist.

Patients were discharged shortly after the colonoscopy. A telephonic visit was performed two weeks after the procedure to collect symptoms related to possible complications. Patients were asked in a direct form about symptoms and if they required additional medications or medical consultation.

### 2.4. Performance Assessment

The optical video from colonoscopy was carefully examined and temporally segmented into sets of consecutive and homogeneous frames. A total of 73 sections were identified, with an average duration of 55 s (45 min 21 s in total), corresponding to approximately 13,600 frames. Of these sections, 23 (31.5%) contained a polyp, 19 (26%) water, 2 (2.7%) angulations, 1 (1.4%) debris and 28 (38.4%) were classified as healthy and clean colon sections.

The same temporal segmentation was performed in the reconstructed synthetic image of the dielectric contrast profile to facilitate the identification of features. Using frames containing endoscopic images of polyps as the ground truth, the segments of the synthetic image reconstruction were carefully examined and the detections were classified as true positives (TPs), true negatives (TNs), false positives (FPs) and false negatives (FNs).

### 2.5. Adverse Events

Adverse events (AEs) were defined following the ASGE lexicon [23] as an event that prevents completion of the planned procedure and/or results in hospital admission or changing of the plan of care. Unplanned events that did not fulfill this definition were considered as incidents.

Adverse device effects not included on the ASGE lexicon but commonly monitored on clinical investigation of devices such as broken or compromised parts, loose or detached parts and usability deficiencies were also collected and analyzed.

A subjective perception of difficulty of the MiWEndo-assisted colonoscopy procedure was made by the endoscopist based on a 5-points Likert scale (very easy to very difficult) including variables such as maneuverability during insertion and retrieval, and difficulties in polyp resection [24].

### 2.6. Outcomes

The primary outcomes were the rate of cecal intubation, number of incidents and AEs, mural injuries and performance metrics for the detection of polyps. Secondary outcomes were the following: patients’ subjective feedback related to the procedure, insertion and procedural time and the perception of difficulty by the endoscopist.

### 2.7. Statistical Analysis

Continuous data were described using means with standard deviation (SD), minimum and maximum. Categorical data were shown as frequencies and percentages. Comparison of continuous data was performed using the Mann–Whitney U test. Performance characteristics for the detection of polyps were calculated using the standard formulas. A two-sided significance level of 5% will be used for confidence intervals. SAS^®^ version 9.4 was used to analyze the data.

## 3. Results

Fifteen patients were enrolled (nine men, six women; mean age, 59.5 years; age range, 51–73). A total of 2/15 (13.3%) had undergone a previous abdominal surgery. Patient preparation was adequate (good or excellent) in all cases. Diverticula were present in 4/15 (26.7%), and all cases were restricted to the sigmoid colon. The adenoma detection rate was 87% (13/15), with a total of 44 polyps (mean 2.9 ± 2.4, range 0–7) with a mean maximum diameter of 4.3 ± 2.7 mm (2–12). Characteristics of the patients are described in Table 1.

### 3.1. Feasibility Results

**Figure 2:**
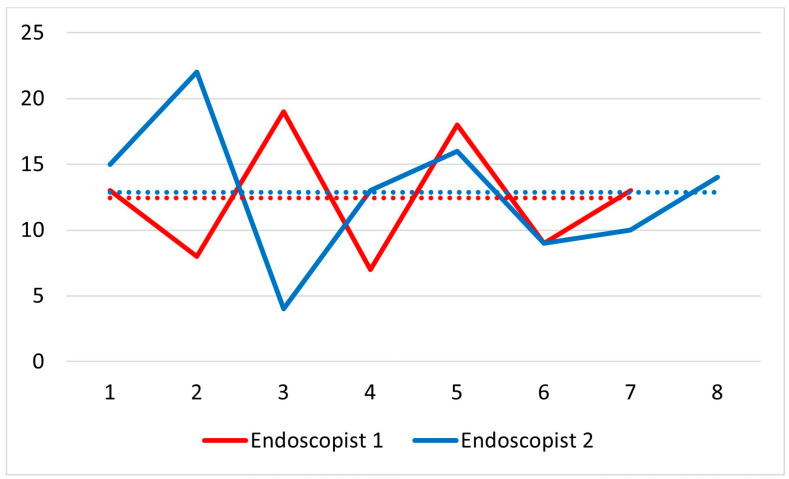
Insertion time of each colonoscopy separated by the endoscopist. The dotted lines represent the median times.

The cecal intubation rate was 100% (Table 1). The mean time to reach the cecum was longer in women than in men (total: 12.7 ± 4.9 min, range 4–22; women: 15.7 ± 4.3 min, 10–22; men: 10.7 ± 4.4 min, 4–18; *p* = 0.048), with a mean total procedure time of 26.6 ± 6.7 min (range 16–40) and a mean withdrawal time of 8.4 ± 3.1 min (range 5–16). Figure 2 shows the insertion time for each colonoscopy separated based on the two endoscopists.

Use of the device did not affect the quality of the colonoscope’s image, and there was no restriction of mobility, tip deflection or retroflexion. Polypectomy was successfully performed in all cases and 39/44 (88.6%) polyps were retrieved for pathological analysis. No dislocation of the device occurred in any of the examinations. On a difficulty scale from 0 (not difficult) to 4 (very difficult), in 86.7% (13/15) of colonoscopies, endoscopists rated the maneuverability during the insertion as ≤2. Two cases had a score of 3 and the difficulty was attributed to a loose sigmoid colon.

### 3.2. Safety

**Figure 3:**
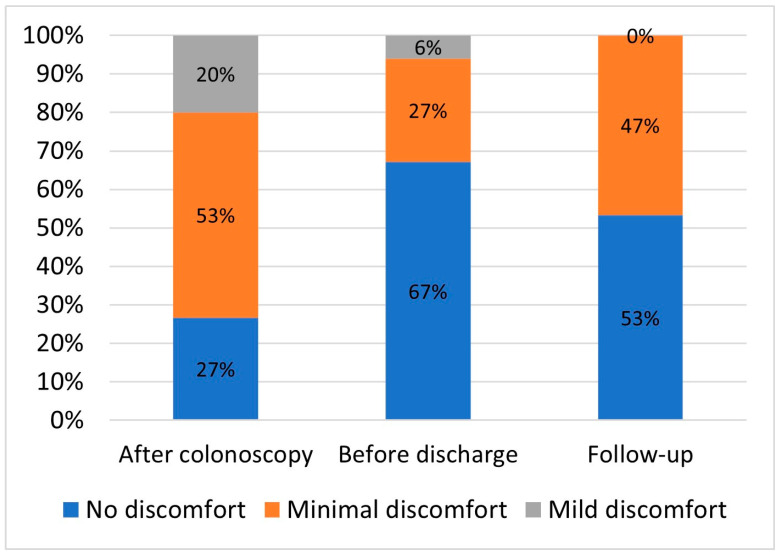
The patients’ mean overall discomfort score.

No immediate or delayed adverse events were recorded. A total of 16 incidents were reported in 14 patients: 11 (67%) superficial hematomas, mainly located at rectosigmoid junction, 2 minor auto limited rectal bleedings, 1 anal fissure, 1 rhinorrhea and 1 headache (Table 2). The patients’ mean overall discomfort score before discharge was 1.4 + 0.6 (range 1–3), and 14 patients (94%) reported no discomfort or minimal discomfort (Gloucester score 1 and 2, respectively) (Figure 3).

### 3.3. Performance

Six patients with 23 polyps were used for the performance analysis. Processing and analysis were not possible in the other patients due to hardware issues (cable disconnections and loss of water-tightness) in four patients, lack of video or pathology analysis for ground truth extraction in two patients, and the absence of polyps in three patients. Table 3 shows the main characteristics of each processed patient and polyps. Of these, 16 (69.6%) were adenomas with LGD, 6 (26.1%) were hyperplastic, and 1 (4.3%) was a serrated sessile polyp.

The sensitivity and specificity for polyp detection were 86.9% and 72.0%, respectively (Table 4). When including only adenomatous polyps, sensitivity increased to 93.7%. A total of 14 false positives were recorded, the majority (78.6%) caused by the presence of water accumulations or debris, while the remaining cases were due to deep angulations. Regarding false negatives, three polyps were not detected by MiWEndo system: two 2 mm, slightly elevated hyperplastic polyps and one 8 mm sessile adenoma located within two folds.

**Figure 4:**
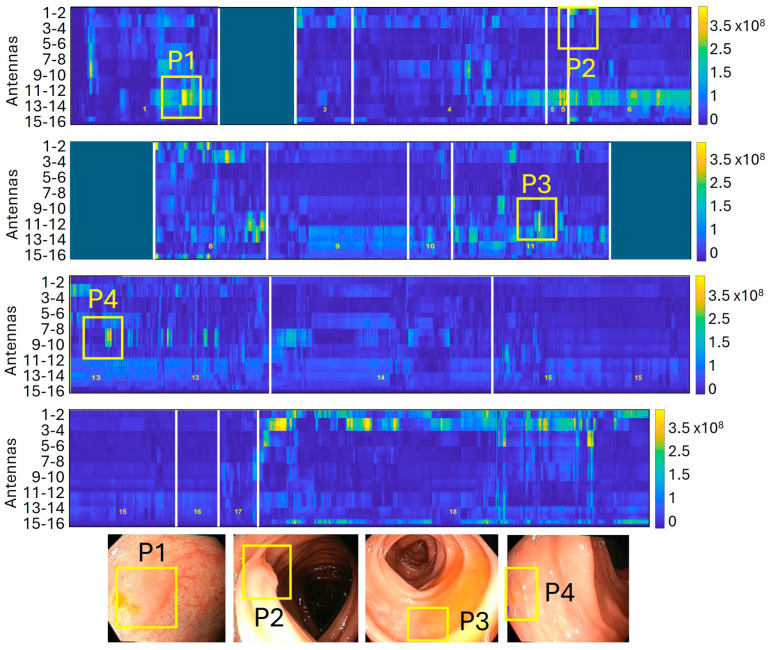
Reconstructed synthetic dielectric contrast image composed of 18 sections with a total duration of 16 min. The image displays low-contrast areas in blue, corresponding to healthy tissue, and high-contrast areas in yellow, which are identified as positive detections. In this trajectory, four adenomatous polyps measuring 10, 8, 5, and 2 mm were correctly detected, with no false negatives.

Figure 4 shows a reconstructed synthetic dielectric contrast image of a colonoscopy containing four adenomatous polyps.

## 4. Discussion

This study reports the first clinical experience with a new concept of colonoscopy based on microwave imaging for polyp detection and shows that it is feasible, safe and has good performance. The MiWEndo System is the only clinically validated microwave endoscopy system that seamlessly integrates into standard colonoscopy without altering clinical practice. It employs low-power radio-frequency signals to examine the patient’s colon, without causing discomfort to the patient or hindering or distracting the endoscopist. So far, this technology has demonstrated its diagnostic capability in phantom ex vivo human colons and in vivo animals [17,18,19].

Other accessory devices have been developed to increase the endoscopes’ field of view [24,25,26]. Artificial intelligence (AI) is also being explored as a tool for automatic polyp detection in colonoscopy, utilizing machine learning and deep learning algorithms that analyze the optical colonoscopy video feed in real time, identifying variations in texture, shape, and color associated with different types of lesions [27]. While AI has demonstrated an increase in the adenoma detection rate (ADR) in most of the studies [7,8,9], its effectiveness is inherently limited to polyps visible within the optical camera’s field of view. Polyps with specific features (i.e., isochromatic, flat, and unclear boundaries), polyps for which the view is partly blocked and polyps that are on the edge of the visual field are easy to miss by endoscopists, but also by AI [28]. Contrarily to other technologies that cannot see what is not shown in the image, MiWEndo can differentiate between healthy mucosa and neoplastic lesions based on the dielectric properties contrast, thereby complementing the endoscopic image. In a previous study, we showed that the dielectric properties correlate with the malignancy and grade of dysplasia of colorectal polyps, and we did not find significant differences in dielectric properties due to the shape of the polyps [15]. Polyps located behind mucosa folds also represent a significant percentage of missed lesions, due to the limited field of view of current endoscopes. As a result, a device capable of scanning the mucosa in a 360° range and processing each frame in 200 milliseconds could help to address this issue in real-time explorations. Conversely, polyps that are very small in diameter appear to be more challenging to detect with microwaves, where AI-assisted optical imaging could provide valuable support. Although not used in this experiment, our intention is to emit an acoustic signal to alert based on the presence of a polyp and allow the endoscopist to be focused and concentrated on the standard endoscopic image. This makes a big difference compared to other existing devices that use artificial intelligence which depict boxes in the screen [29], or side-viewing endoscopes that display the image on one or two accessory screens [30].

The system was designed to be compatible with all types of colonoscopes, ensure a 360° field of view and produce minimal changes to the current clinical practice. The dimensions and shape of the device ensure non-obstruction of the camera and avoid hindering the maneuverability of the colonoscope, even during therapeutic procedures, such as polypectomy. In this trial, we found a cecal intubation rate of 100%, indicating a high effectiveness of the microwave-colonoscopy in terms of completeness of examination, even in patients with diverticula and previous abdominal surgeries. However, we decided to exclude patients in whom the possibility of performing a complete colonoscopy was reduced, and these preliminary results must be interpreted with caution because of the non-randomized design with a small sample size. Moreover, all the colonoscopies were performed at a highly specialized endoscopic center by expert endoscopists after a specific training.

The cecal intubation time was longer compared to standard colonoscopy and colonoscopy with other accessory devices [23,24,25]. This is more likely because of the sleeve and the transmitter cables than the size of the cap. Moreover, during early testing, endoscopists were not as familiar with the device, and there was a tendency to apply less pressure during insertion. As they gained confidence, the pressure exerted also increased, although this did not translate into a higher insertion speed, most likely due to patients’ anatomical differences and the small sample size. In the two patients in whom the intubation was very difficult, it was due to a “loose sigmoid” and the opinion of the endoscopist was that the colonoscopy would have been as difficult even without the use of the accessory.

Notably, the system achieved a high sensitivity of 86.9% for polyp detection when considering all polyp types. Even more interestingly, sensitivity increased to 93.7% when only adenomas were included. This occurs because hyperplastic polyps are generally small and have no dielectric contrast with healthy colon tissue, making them very difficult to detect using microwaves. From a clinical perspective, this is particularly relevant because small hyperplastic polyps in the sigmoid and rectum can be left untreated without requiring resection following the ‘diagnose and leave’ strategy. According to the thresholds set by the American Society for Gastrointestinal Endoscopy (ASGE), a NPV of more than 90% for adenomatous histology is recommended to support it [31]. Since the NPV for the MiWEndo System was 97.3%, it seems that it could potentially support this approach by providing additional diagnostic information beyond standard optical imaging.

Regarding specificity, the majority of false positives were caused by water accumulation due to the high dielectric contrast between water and healthy colon tissue, which was even greater than the contrast between healthy colon tissue and polyps or cancer [16]. The effect of water on the microwave image has a different signature compared to polyp detection: while a polyp presents a short-duration detection, water causes a much more prolonged effect and always appears in front of the same antenna combination. Therefore, we are developing new automatic detectors based on neural networks [32] that can leverage different temporal patterns and distinguish polyps from other types of noise, as these have different temporal patterns. The effect of stool on the microwave signal could not be precisely assessed, as any presence of stool was diluted with water to enhance optical visualization. The only instance where the effect could be analyzed was in a section containing small stool remnants without water accumulation, where no false positives were detected. The influence of stool on detection should be specifically investigated in future studies.

The main concern regarding the use of an accessory device that not only increases the size of the endoscope but also its stiffness due to the presence of connecting cables and the sleeve is an anticipated higher perforation rate, as can be seen with long overtubes [33]. In this trial, no adverse events were recorded. Among the incidents, in all patients except two, superficial hematomas located at the rectosigmoid level and/or rectum were seen at the end of endoscopy without any clinical symptoms. These lesions were expected and were most likely caused by friction of the sleeve with the cables inside. However, similar lesions are commonly observed in standard colonoscopy procedures and when using other add-on devices [34]. Because of the larger caliber of the tip of the colonoscope compared to standard colonoscopes, anticipated strictures might be a contraindication for microwave-assisted colonoscopy.

The main limitation of this study is the small sample size, both in terms of patients and polyps. However, for the evaluation of medical devices, there is an initial stage that should include a low number of patients as stated by the IDEAL Framework and Recommendations [35]. Due to the limited number of cases, there are several relevant issues, such as the potential patent-specific tissue changes or the effect of inflammation on the dielectric contrast, which have not been addressed yet. Another limitation is that all procedures were performed under deep sedation, in line with our clinical practice. As a result, the assessment of patient comfort is limited. However, patients reported high overall satisfaction on average. Finally, since the explorations were performed by experienced endoscopists, the results of usability cannot be extrapolated to more inexperienced endoscopists.

## 5. Conclusions

In summary, MiWEndo is a microwave-based endoscopy system designed to enhance polyp detection during colonoscopy, aiming to reduce the rate of missed lesions and, consequently, lower the incidence of post-colonoscopy colorectal cancer (CRC). This study demonstrates that the system is safe, feasible, and has the potential to improve polyp detection without altering clinical practice. Larger prospective studies are needed to further evaluate its efficacy in detecting polyps, confirm its safety and assess its true clinical benefit across different settings.

## Figures and Tables

**Table 1:** Patients’ characteristics and colonoscopy data.

Patients (N = 15)	
Gender, N (%)	
-Men	9 (60%)
-Women	6 (40%)
Age, mean ± SD (range), years	59.5 ± 6.7 (51–73)
Indication for colonoscopy, N (%)	
-Surveillance after polypectomy	9 (69.2%)
-Abdominal pain	3 (23.1%)
-Lower GI bleeding	1 (7.7%)
Boston scale, mean ± SD (range)	7.9 ± 1.3 (6–9)
Cecal intubation rate, N (%)	15 (100%)
Time to cecum, mean ± SD (range), minutes	12.7 ± 4.9 (4–22)
Withdrawal time, mean ± SD (range), minutes	8.4 ± 3.1 min (5–16)
Total procedure time, mean ± SD (range), minutes	26.6 + 6.7 (16–40)
Adenoma detection rate, N (%)	13 (87%)
Total polyps per patient, mean ± SD (range), N	2.9 ± 2.4 (range 0–7)
Histology	
-Adenoma with LGD, N (%)	28 (60.9%)
-Hyperplastic, N (%)	10 (21.7%)
-SSL, N (%)	1 (2.2%)
-Not retrieved	7 (15.2%)

American Society of Anesthesiologists; SSL, serrated sessile lesion.

**Table 2:** Incidents and adverse events in study of the feasibility and safety of colonoscopy assisted by microwave imaging.

Incidents	Frequency	Related to MiWendo
Mucosal abrasions, N (%)	11 (73.3%)	Yes
Bleeding, N (%)	2 (13.3%)	Yes
Anal fissure, N (%)	1 (6.7%)	Yes
Headache, N (%)	1 (6.7%)	No
Rhinorrhea, N (%)	1 (6.7%)	No

**Table 3:** Characteristics of the processed patients and polyps detected with the MiWEndo system.

Patient	Number of Polyps	Size (mm)	Morphology	Histology	Location	Detected
1	1	4	0-IIa	SSL	SF	Yes
2	8	6	0-IIa	Adenoma	AC	Yes
		2	0-IIa	Adenoma	AC	Yes
		2	0-IIa	Hyperplastic	TC	No
		3	0-IIa	Adenoma	TC	Yes
		2	0-IIa	Hyperplastic	TC	Yes
		4	0-IIa	Adenoma	DC	Yes
		3	0-IIa	Adenoma	DC	Yes
		10	0-IIa	Hyperplastic	SC	Yes
3	5	3	0-Is	Adenoma	AC	Yes
		4	0-IIa	Adenoma	AC	Yes
		8	0-Is	Adenoma	HF	No
		12	0-IIa	Adenoma	TC	Yes
		2	0-IIa	Adenoma	TC	Yes
4	4	10	0-IIb	Adenoma	C	Yes
		8	0-IIa	Adenoma	TC	Yes
		5	0-IIa	Adenoma	SC	Yes
		2	0-IIa	Adenoma	SC	Yes
5	1	3	0-IIa	Hyperplastic	TC	Yes
6	4	2	0-IIa	Hyperplastic	AC	Yes
		2	0-IIa	Hyperplastic	HF	No
		3	0-IIa	Adenoma	TC	Yes
		4	0-IIa	Adenoma	SC	Yes

SSL, serrated sessile lesion; SF, splenic flexure; AC, ascending colon; TC, transvers colon; DC, descending colon; HF, hepatic flexure; C, cecum; SC, sigmoid colon.

**Table 4:** Performance metrics of polyps’ detection with MiWEndo System.

	All Polyps N = 23	Adenomas N = 16
True positives	20	15
False positives	14	14
True negatives	36	36
False negatives	3	1
Sensitivity	86.9%	93.7%
Specificity	72%	72%
PPV	58.8%	51.7%
NPV	92.3%	97.3%

## Data Availability

The authors provide no restriction on the availability of the methods, protocols, instrumentation and data utilized in this article. Data are available from the corresponding author upon reasonable request.

## Abbreviations

The following abbreviations are used in this manuscript:

CRC	Colorectal cancer
MWI	Microwave imaging
TP	True positives
TN	True negatives
FP	False positives
FN	False negatives
AE	Adverse events
AI	Artificial intelligence
ADR	Adenoma detection rate
ASGE	American Society for Gastrointestinal Endoscopy
